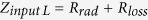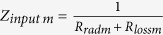# Pushing the limits of radiofrequency (RF) neuronal telemetry

**DOI:** 10.1038/srep10588

**Published:** 2015-06-02

**Authors:** Tara Yousefi, Rodolfo E. Diaz

**Affiliations:** 1 School of Electrical, Computer and Energy Engineering, and Security & Defense Systems Initiative, Arizona State University, Tempe, Arizona, 85287 USA

## Abstract

In a previous report it was shown that the channel capacity of an *in vivo* communication link using microscopic antennas at radiofrequency is severely limited by the requirement not to damage the tissue surrounding the antennas. For dipole-like antennas the strong electric field dissipates too much power into body tissues. Loop-type antennas have a strong magnetic near field and so dissipate much less power into the surrounding tissues but they require such a large current that the antenna temperature is raised to the thermal damage threshold of the tissue. The only solution was increasing the antenna size into hundreds of microns, which makes reporting on an individual neuron impossible. However, recently demonstrated true magnetic antennas offer an alternative not covered in the previous report. The near field of these antennas is dominated by the magnetic field yet they don’t require large currents. Thus they combine the best characteristics of dipoles and loops. By calculating the coupling between identical magnetic antennas inside a model of the body medium we show an increase in the power transfer of up to 8 orders of magnitude higher than could be realized with the loops and dipoles, making the microscopic RF *in-vivo* transmitting antenna possible.

In a previous report the electromagnetic limits to radiofrequency (RF) telemetry from within the brain, have been discussed. By establishing a link budget consistent with picowatts of radiated power at the surface of the head, it was shown that to avoid thermal damage to tissue surrounding the transmitting embedded antenna, and still be able to communicate at 300 Kbps with a reasonable link margin from within the human brain, electric dipoles at least 680 μm in length or magnetic dipoles at least 59 μm in diameter would be required. For the rodent brain case these numbers become 250 μm and 26 μm respectively. In the rodent case, it was estimated that a 14.5 μm diameter loop antenna with a magnetic core (that raised its dipole moment by a factor of 3) could communicate at the reduced rate of 3 Kbps. Making the antennas any smaller increases the power dissipated into the proximate tissues above the accepted safety limits^1^. Assuming that the desired size of a sufficiently unobtrusive embedded telemetry node (antenna plus on-board transmitter) is 10 μm, these results rendered unfeasible the prospect of using such a microwave RF telemetry system to track neuronal activity *in-vivo*^2^,^3^,^4^.

The root of the problem is that small electric dipole antennas embedded in lossy dielectric media suffer very large near field loss in the process of radiating a signal and loop antennas suffer excess Ohmic loss in their metal elements. Therefore when these antennas are fed sufficient power to transmit through the brain, the electric dipole would damage the nearby tissue through specific absorption rate (SAR) deposition and the electric loop antenna through heat conduction. But this is not the end of the story.

In the reference paper^1^, the effect of a magnetic core for loop antennas was only modeled as an increase of the antenna’s dipole moment thus leading to a slight benefit in radiated gain. The reality, evident from recent work on magnetic conformal antennas^5^,^6^,^7^ is that the magnetic core of a loop antenna can dramatically alter its input impedance, changing its behavior from that of a metal loop to that of a permeable dipole. In the regime where the magnetic core dominates the behavior, the principal radiating current is not the electric current in the loop but the magnetic polarization current in the core material. Because of the high efficiency properties of these magnetic dipoles, when located in a low impedance environment, have not been reported until recently^5^,^6^,^7^ we describe them below before proceeding with their application to the *in vivo* neuronal telemetry scenario.

## True Magnetic Antennas

Although in the conventional practice of Antenna Theory and Design a distinction is made between electric dipole antennas (generally metal rods carrying an alternating electric current) and magnetic dipole antennas (generally metal loops carrying an alternating electric current,) in reality both of these antennas are electric current radiators. In the first, the electric current is linear and couples most efficiently to the electric dipole modes of the spherical mode spectrum; in the second the electric current is circumferential and couples most effectively to the magnetic dipole modes of the spherical mode spectrum. Since the work of O. Heaviside^8^ in the late 1880’s it has been known that Maxwell’s equations also admit of the presence of magnetic currents. Therefore, behaviors equivalent to magnetic current radiators exist in principle.

Most antenna practitioners assume that the absence in nature of observed magnetic monopoles precludes the existence of true magnetic currents and therefore whenever the term magnetic dipole is used, a loop antenna is usually meant, and whenever the term magnetic current is used, a fictitious magnetic current is usually meant. The latter currents arise in Schelkunoff’s Equivalence Theorem whenever it is desired to summarize all the sources on one side of a closed mathematical surface by using the tangential electromagnetic fields existing on that surface. Thus the surface fictitious magnetic current, Km (measured in Volts/meter) is defined as the cross product of the surface normal and the tangential Electric field on that surface.

However, in this paper we are talking about true (as opposed to fictitious) magnetic current radiators. That is, in the same way that electric current density, J_e_, (measured in A/m^2^) flows through a medium with electric conductivity, σ_e_ (measured in Siemens/meter), as:
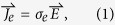


Magnetic current density, J_m_, (measured in V/m^2^) flows through a medium with magnetic conductivity, σ_m_ (measured in Ohms/meter), as
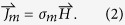


Even though magnetic conductivity does not exist at zero frequency (because of the absence of magnetic monopoles), as far as Maxwell’s equations are concerned, it exists at any frequency in which a material exhibits a magnetic loss tangent. This is because the imaginary part of the complex permittivity and complex permeability of materials, *ε*(*ω*) = *ε*_0_(*ε*′ − *jε*″), and *μ*(*ω*) = *μ*_0_(*μ*′ − *jμ*″), imply the existence of a corresponding conductivity through the relations:
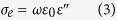

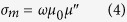


Thus in Maxwell’s curl equations; the terms involving the rate of change of the flux densities imply the existence of magnetic currents as well as electric currents as shown below:
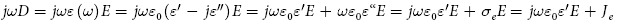

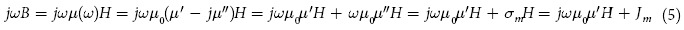


**Figure 1:**
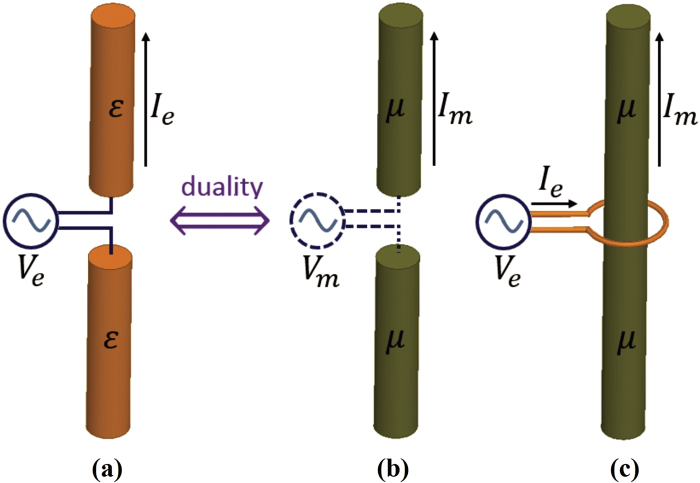
Electric dipole carrying an alternating electric current and its electromagnetic dual which is the magnetic dipole (in this case modeled as rods).

Therefore, whenever a lossy permeable material is used to carry an alternating magnetic field, it behaves exactly as if it were carrying a magnetic current measured in Volts (whereas electric current is measured in Amps) as suggested in Fig. 1.

Figure 1 shows an electric dipole carrying an alternating electric current and its electromagnetic dual which is the magnetic dipole (in this case modeled as rods). Duality requires that since the electric dipole has Perfect Electric Conductor (PEC) feed lines and an electric voltage source load (*V*_*e*_), the magnetic dipole should have Perfect Magnetic Conductor (PMC) feed lines and a magnetic voltage source (*V*_*m*_). Since there is no access to PMC feed lines and magnetic voltage sources, a PEC feed loop is used instead to feed the magnetic dipole^6^.

Accordingly, throughout this paper the term electric loop refers to a metal loop with no core, electric dipole refers to our copper sphere model of a linear conventional electric dipole, and the true magnetic antennas just defined, will be referred to as magnetic dipoles.

The impact this difference makes in the results of the previous study^1^ can be understood in its simplest terms by comparing the two magnetic dipole radiators, one the conventional electric loop antenna, the other, the same loop antenna wrapped around a sphere of dispersive ferromagnetic (or ferrimagnetic) material and comparing their input resistance and radiation efficiency at resonance.

### Comparison of the true magnetic dipole to the conventional loop

**Figure 2:**
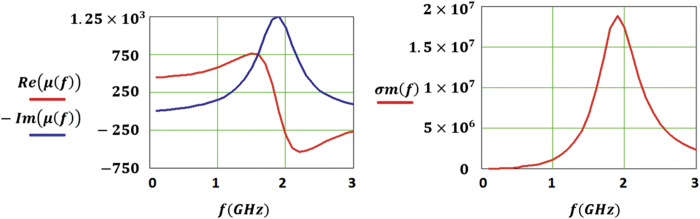
Relative permeability (left, unitless) and corresponding Heaviside magnetic conductivity (right, in Ohms/meter) of a typical high frequency ferromagnetic material (*μ*_*initial*_ = 450).

To highlight the difference in performance between the true magnetic dipole and a conventional loop antenna it suffices to compare them in free space. The magnetic core will be assumed to be a laminated ferromagnetic material like those used in the magnetic read-head industry. The typical frequency dependent permeability of a single metal layer of such a material (CoZrNb, abbreviated CZN, thin film^9^) is shown Fig. 2. Heaviside’s magnetic conductivity is defined as *σ*_*m*_ = *ωμ*_0_*μ*″, where μ_0_ is the permeability of free space and μ” is the imaginary part of the relative permeability.

Assuming the electric loop antenna is a copper loop of conductivity *σ*_*Cu*_ = 6 ⋅ 10^7^*S*/*m*, radius *a* = 10μm and wire cross sectional radius ρ = 0.5μm. The relevant parameters for both antennas can be approximated as shown in Table 1.

Since the radiation efficiency of an antenna is the ratio of its radiation resistance to its total resistance, at 1.5 GHz (the resonance peak of the permeable core) we find that the efficiencies of the electric loop and the magnetic dipole are:
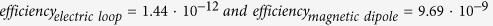


As expected the efficiencies of these microscopic antennas are very small, but that of the magnetic dipole is over 6500 times greater than that of the electric loop antenna. At the same time, *Z*_*input*_*L*_ = 1.33Ω while *Z*_*input*_*m*_ = 174Ω. The magnetic dipole has a much higher input impedance than the electric loop. Therefore while the electric loop must draw a large current to radiate its signal (and thus gets very hot), this magnetic dipole instead develops a high voltage while radiating its signal (much like conventional electric dipoles).

Since we know from the previous study that near field SAR deposition into the surrounding tissue is not a concern for magnetic dipoles the only damage risk here comes from raising the antenna temperature. But because the true magnetic dipole has a much higher impedance than the conventional loop antenna it follows that to radiate equivalent power it will draw a much lower current and thus dramatically reduce the risk of damage through heat conduction.

The end result of these considerations is that the link budget calculation performed in the original report must be redone for this new type of antenna. However, that calculation will be left to a future third report in this series because there is a more important development that results from choosing microscopic magnetic dipoles as candidate *in-vivo* RF telemetry antennas. The benefits highlighted by the calculations above in free space pale in comparison to the benefits derived inside the dielectric lossy medium of the body, especially if we also assume that the receiving antenna is a magnetic dipole itself.

In the first report it was seen that the best case human subject scenario requires the receiving antenna to be located just outside the head. Of course any antenna in close proximity to the head will still be affected by the lossy properties of the brain, given the extent of penetration of the antenna’s near field. This point was not belabored in that report because accurate modeling of reasonable external antenna configurations would not have changed the essence of the pessimistic results obtained. However, in light of the potentially optimistic results that magnetic dipoles might bring, it is important to consider this effect. And we do so as the worst case scenario where the receiving antenna is itself assumed to be completely immersed in the same lossy dielectric medium as the transmitting antenna.

Therefore, for the balance of this report we concentrate on calculating the signal received from a microscopic antenna by a second microscopic antenna of the same kind in the same medium. The calculation is simplified by assuming (as in the first report) reasonable simple models of the antennas that allow the derivation of a closed-form expression for the mutual coupling between the antennas. The efficiency of signal transmission is then simply expressed as the ratio of the current induced on the receiving antenna to the current driving the transmitting antenna. Since received power is proportional to the square of the antenna current, the gain in efficiency is proportional to the square of this ratio.

### The case of the insulated Electric Dipole

Before proceeding with this comparison it is worth mentioning the case of the insulated electric dipole, a case also not addressed in the original report^1^. Long linear electric dipoles are commonly used for communication under sea water, an extreme case of a low impedance medium. Because of the obvious conductivity of seawater medium, it is to be expected that these dipoles should be insulated from the medium to prevent the medium from shorting them out. And the question then arises, should not electric dipoles inside the body also be insulated? The answer can be found in in Kraichman’s monograph^10^. Although electric dipoles are insulated for most of their length under seawater, they only attain maximum gain if their ends are electrically connected to the conducting medium. If they are completely insulated, instead of an antenna we have a center fed coaxial cable, with the antenna as the inner conductor and the seawater boundary as the outer conductor. The wave injected at the feed of the dipole remains trapped in this coaxial line, reflecting back and forth between its ends.

The same effect arises in the case of our model spherical antenna immersed in the lossy body dielectric, except in our case the ends of the dipole are the conducting hemispheres and the insulated length is the assumption that the feed region itself is insulated from the medium. That is, our closed-form model of the electric dipole as a spherical antenna already behaves as if a narrow strip of insulator were wrapped around the equator where the voltage is applied to the hemispheres. If we were to insulate any portion of the hemispheres themselves, the result would be to add a shunt capacitance at the feed that diverts part of the feed current back to the source, thus reducing the amount of radiating current and therefore the gain. At the same time the loss resistance presented to the antenna by the outer medium, contacting through a smaller area of the hemispheres, would be increased.

If we suppose that the entire antenna were insulated by a very thin dielectric layer of thickness of t_L_, then it is true that we have removed a layer of thickness t_L_ from the near field ohmic loss integration because now the integral does not extend from a to ∞ but instead from a + t_L_ to ∞. However, this would happen automatically if we had increased the radius of the dipole by the same thickness t_L_. So the change in near field loss due to a change in radius is not a relevant comparison.

Suppose we keep the outer radius constant and decrease the metal sphere’s radius by inserting the insulating layer. The biggest impact of the insulating layer is that we have effectively placed the dipole at the center of an insulating dielectric cavity inside the conducting body medium. Now the input impedance of the small spherical dipole is given by putting in series with the external capacitance, the capacitance of the insulating layer. This capacitance reduces the antenna’s total capacitance, therefore raising its capacitive reactance, and requiring now a larger inductor to resonate it. But that’s not the main problem. Some flux lines from the upper hemisphere do terminate on the cavity wall while some flux lines terminate on the lower hemisphere. Only those flux lines terminating on the wall induce charges on the surface of the body boundary and it is only those oscillating charges that constitute the dipole moment radiating into the body. The field lines not terminating on the wall simply constitute a shunt capacitance. Thus for a given input voltage we end up reducing the radiated power and increasing the stored energy. The only thing this accomplishes is decreasing the bandwidth of the antenna, and all the near field loss from a to ∞ is still there.

Thus, the original conclusion from the first report^1^ still holds: antennas operating in the magnetic dipole mode have a decided advantage. We show in this contribution that two identical microscopic true magnetic dipole antennas can communicate inside the lossy dielectric body medium with an efficiency that is over 8 orders of magnitude greater than could be attained by conventional electric dipoles or electric loop antennas of the same size. It appears that microscopic RF telemetry inside the brain is now feasible.

## Results

It is known that micron sized antennas store much more energy in their near field per cycle than the power they can radiate to the far field by a ratio called the Quality factor of the antenna. The Fano-Chu limit sets a lower limit^11^,^12^,^13^,^14^,^15^ for this ratio which can be approximated by *Q* ≈ 1/(*ka*)^3^, where *k* is the propagation constant of the medium and *a* the radius of the smallest sphere that can enclose the antenna. For example if the body were considered a lossless dielectric, a 20 micron antenna inside the body at 2 GHz, would have a minimum Q of 40 million and an 80 micron antenna at the same frequency would have a Q of the order of 600,000. Since the body is a lossy dielectric, these Q values are reduced in actuality but they still serve to gauge the amount of energy per cycle stored in the near field, energy that is then consumed as heat. Therefore to produce a reasonable radiated power outside the head (in the picowatts range) it was found in our previous analysis^1^ that microscopic antennas must be supplied with microwatts of power.

The baseline assumption made in the analysis of reference 1 is that one isolated antenna tries to communicate to the outside world by direct radiation of its signal. Furthermore, in that analysis the effect of adding a magnetically permeable core to a loop antenna was only modeled as an increase in the dipole moment of the loop by at most a factor of 3. The effect on the input impedance of the loop antenna and the possibility that the main radiating current could be the polarization current of the core, and not the electric current of the loop, were not considered. Yet, recent developments in the theory and practice of magnetically permeable antennas^15^,^16^,^17^ have shown that such antennas exhibit unprecedented gain and efficiency in the presence of a low impedance environment. The result is that a conformal magnetic antenna (constructed from a lossy ferrite) only 1.5 inches thick, lying directly on the conducting roof of a High Mobility Multipurpose Wheeled Vehicle (HUMVEE), was shown to outperform an eight foot tall conventional metal monopole antenna on the same vehicle^5^. Since complete immersion in a lossy dielectric also presents a low impedance medium to an antenna, it can be expected that microscopic magnetic antennas inside the body dielectric will also show unprecedented gain and efficiency. This is demonstrated by calculating the mutual coupling between two identical electric dipole antennas, magnetic dipole antennas and electric loop antennas immersed in the body dielectric.

From the impedance point of view, electric loops and electric dipoles are the two fundamental types of antennas. According to Schelkunoff^16^, although in order to calculate the impedance of an antenna we might have to solve Maxwell’s equation subject to the specific boundary conditions of the antenna, we can obtain some of the important general properties of the impedance from much more basic considerations. An interesting fact is that these properties are not limited to antennas or electrical systems. They are common to all dynamic systems (for example mechanical and acoustical) and they don’t even depend on the form of the dynamical equations as long as those equations are linear. A brief consideration of these general properties of impedance will help to understand the reason for the difference in the antennas’ behavior.

For any transmitting antenna the voltage and current at the input terminals can be written as a function of a complex variable which can be called “p” where *p* = *jω* and *ω* is the frequency of oscillation. The ratio of the functions V(p) and I(p) is called the input impedance Z(p) of the antenna and the inverse is called the input admittance Y(p).

**Figure 3:**
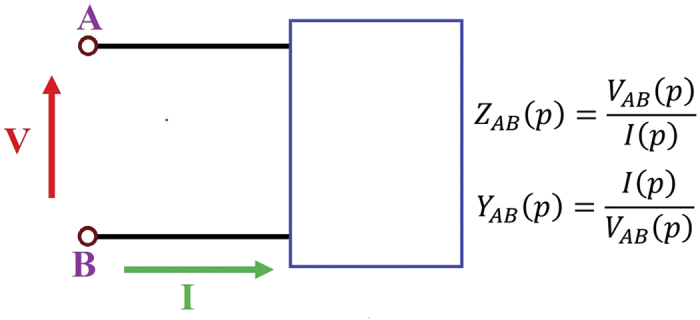
A schematic of a network with two accessible terminals.

The schematic in Fig. 3 shows a network with two accessible terminals and in order to introduce the two fundamental antenna types we can write the impedance and admittance as stated in the figure without being interested in their interior structure.

The roots of the equation *Z*(*p*) = 0 are called the zeros^16^ of the input impedance, which are the cases for which the voltage across the input impedance vanishes while the current does not. The poles of the impedance are the zeros of the admittance and are roots of the equation (*p*) = 0, and it is obvious that in this case the input current goes to zero while the voltage does not, which means that the terminals of the antenna are floating or the antenna is an open circuit.

**Figure 4:**
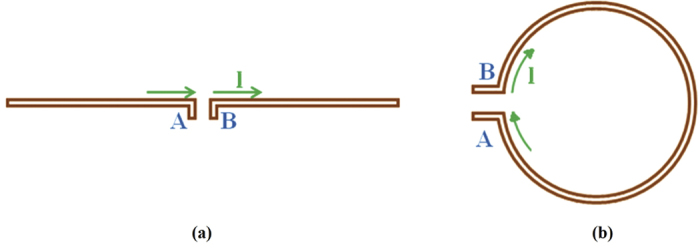
Two general types of antennas. (**a**) dipole antenna (**b**)loop antenna

If the terminals of an antenna are open circuited conductors, we can place opposite charges on these conductors and create a voltage across the input impedance and the current will be zero, therefore having p = 0 as a pole of its input impedance. These antennas are called dipole antennas. On the other hand an antenna consisting of a single perfect conductor having a steady current flowing in it would not have loss. These antennas are called electric loops and p = 0 is a zero of the impedance of a perfectly conducting electric loop. Figure 4, shows the electric dipole and the electric loop as the two general types of antennas.

This fundamental difference between electric dipole antennas and electric loop antennas is carried through to the case of realistic imperfect environments and materials. The open circuit nature of an electric dipole immersed in a lossy conducting dielectric means that the large voltage developed between its opposite terminals drives a current directly in the medium surrounding it, following the field lines of its near electric field and depositing power into the medium. The short circuit nature of the electric loop means that it cannot drive a current directly in the surrounding medium. Instead currents are induced via electromagnetic induction of eddy currents by its magnetic near field. As shown in reference 1 this results in the loop dissipating much less power into the surrounding medium; but the fact that Copper is not a perfect conductor means that the current in the electric loop will cause it to heat up and dissipate power into its own materials. Given these two different behaviors we could ask if there is another kind of antenna that can combine the best features of these, one whose near field is dominated by a magnetic field and yet does not behave as a short circuit to draw large currents. This is precisely what permeable magnetic dipoles do.

**Figure 5:**
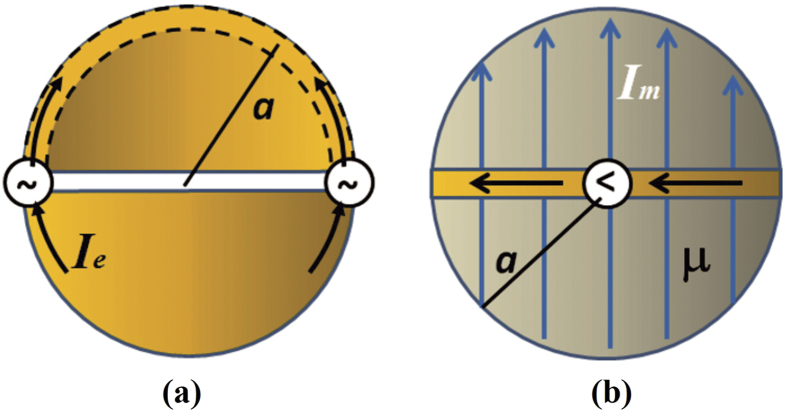
Sketch of the idealized antennas considered in this analysis. (**a**)electric dipole antenna (**b**)magnetic dipole antenna

For the sake of the computational examples we assume spherical antennas 10 *μ*m in radius^5^. (The derivations are included in the Methods section to enable the reader to replicate these results.) Fig. 5 is a sketch of the idealized antenna models represented by these dipoles. We have chosen the sphere geometry as a convenient form for the dipole antennas for many reasons. The spherical geometry of the dipoles makes the radiated field and the mutual coupling calculations straight forward because they can be written in a self-consistent closed form. As we will see later, by using spherical dipoles, we can normalize the radiated field to the field on the surface of the spheres and we can also normalize the induced current to the source current, thus enabling us to compare electric and magnetic antennas on equal footing.

The electric dipole can be imagined as a hollow sphere cut in half and fed by a distributed electric voltage source, V_E_, at the equator such that the total electric current flowing depends on the self-impedance of the antenna (measured in Ohms) according to equation 6.
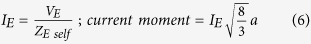


The magnetic dipole can be imagined as a solid permeable sphere with a conducting belt around its equator, said belt fed by a current source^5^,^6^,^7^. The current flowing through the belt in Amps is the magnetic Voltage, V_M_, and the total magnetic current flowing depends on the magnetic self-impedance of the antenna (measured in Siemens) according to equation 7.
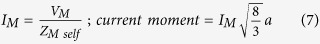


The complete duality in Maxwell’s equations that is evident once magnetic currents were introduced by O. Heaviside^8^, allow the engineer to translate conventional results of electrically conducting antennas driven by an electric voltage at a gap into the results for magnetically conducting antennas driven by a current flowing in a metal feed loop surrounding the permeable material.

Conventional metal antennas have an impedance given by the ratio of the applied voltage, V, to the current that flows in the metal, Ze measured in Ohms. The current, I, can be measured by performing the circulation integral of Ampere’s law around the metal wire, that is
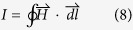


In the same way, permeable antennas have a dual magnetic impedance, Zm, measured in Siemens, given by the ratio of the applied Current, I, in the feed loop to the electromotive force around the permeable rod
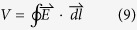


This is why Zm has the inverse units of Ze. In the referenced dissertation^6^, it is shown that Zm is nothing but the electric admittance, Ye in Siemens, measured by the source driving the current in the permeable antenna’s feed loop.

In an ensemble of spheres, the mutual coupling between spheres is represented by the mutual impedance. For electric and magnetic dipoles located on the same x-y plane, all polarized along the z-axis, these are given by equations 10 and 11.
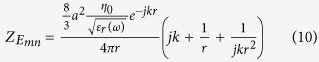

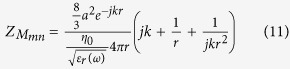


Z_mn_ is the mutual impedance between the m^th^ and n^th^ sphere. Setting r = *a* (the radius of the sphere) yields Z_mm_, the self -impedance. (See the Methods section for the details.) With this formulation it is straight-forward to solve self-consistently the problem of the excitation of an ensemble of spheres by any incident field, or in particular, by one member of the ensemble. Focusing on the simplest case of two spheres, the Voltage at the feed of each antenna depends on the currents on itself and the other antenna, satisfying an equation of the form:
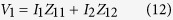


Thus, under the assumption that the two spheres share the same equatorial plane, the problem of an array of two spheres is represented by a matrix equation of equation 13, where all the terms of the Impedance matrix, 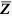, are known from equations 10 and 11.
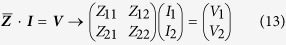


To maximize the power transfer from the source to the antenna (and from the antenna to its receiver) we assume that the antennas have been tuned to resonance at the operating frequency either by aid of a matching circuit or the natural resonance of its constitutive materials. This simply means that the self-reactance has been cancelled.
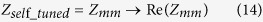


To illustrate how the materials of construction can be used to tune the antenna requires a brief digression. In reference [5] it is shown that, to first order, the input impedance of an electrically small material antenna (as opposed to the idealized perfectly conducting antenna) can be obtained by simply adding in series with the conventional antenna model, the internal Impedance of the material. R. W. P. King and T. T. Wu use a similar argument^18^ to analyze the imperfectly conducting antenna.

In the simplest case of a small metal dipole (our spherical dipole antenna) we can approximate its external impedance by the capacitance of its external near field in series with its radiation resistance. In a lossy dielectric medium the near field capacitance is complex and thus adds extra resistance to the antenna. To resonate (that is to tune) such an antenna the common practice is to add a series inductor such that the series sum of the added inductive reactance and the external capacitive reactance equals zero.

In the same sense a small spherical permeable antenna of radius *a,* has an external impedance dominated by the magnetic capacitance of its near field. Thus its dual magnetic input impedance is approximately
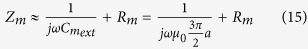


Where *R*_*m*_ is the magnetic dipole’s (dual) radiation resistance. Now, assuming the flux inside the permeable sphere is uniform, the internal capacitance can be roughly approximated by a term of the form:
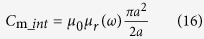


Therefore the total impedance of the material antenna is:
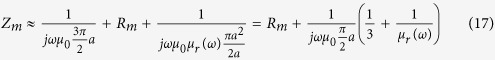


In this last equation we see that the internal and external capacitances add in series and it is clear that when the real part of the relative permeability of the material, *μ*_*r*_(*ω*), gets close to the value -3, the reactance is cancelled and the antenna is resonant. At resonance the input impedance of the antenna is purely resistive, consisting of the radiation resistance, the loss resistance of the antenna metal components and the body dielectric, and the loss contributed by the permeable material’s imaginary part of the permeability. Because a ferromagnetic material having a strong Lorentz-like resonance exhibits negative permeability values just past that resonance it is clear that choosing using such a material for the magnetic dipole’s permeable core can result in an antenna that is automatically tuned by its materials of construction.

Returning now to the calculation of mutual coupling between antennas, in the most general case where the *m*^th^ sphere of an array is excited and the rest are passive, the currents on all antennas are obtained by setting *V*_*m*_ = 1, all other *V*_*n*≠*m*_ = 0 and inverting the matrix:
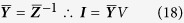


The case of the electric loops is solved similarly and the details of the derivation can be found in the methods section. The final step before solving the case of interest is to define the medium in which the antennas are immersed. As in reference 1, a good approximation below 3 GHz to the FCC accepted model for the human head is a medium of unity relative permeability and relative permittivity given by the following multi-Debye relaxation model including a DC conductivity of 0.68 S/m (with the frequency written in GHz):
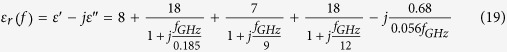


The propagation constant and medium impedance appearing in equations 10 and 11 become:
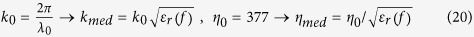


The mutual impedances are then:
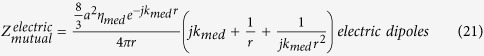

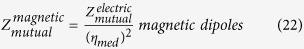

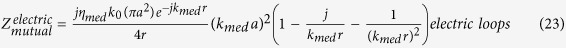


These closed form equations are easy to use and have the pleasing feature that to get the self-impedance we simply set r = *a*. However, as explained above, for the magnetic dipole case the self-impedance has an additional series term^6^ due to the material properties of the core.
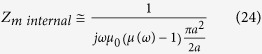


So that for the magnetic dipoles the total self-impedance at resonance is given by equation 25.
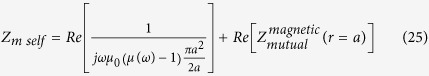


The first term on the left, the real part of the internal impedance, represents the loss inside the permeable core. This turns out to be inversely proportional to the Heaviside magnetic conductivity of the material 
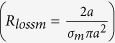
 and thus is minimized when the material has a very large initial permeability (500 in our example) and when the operating frequency is chosen as the resonant frequency of the material. This choice also maximizes the input impedance.

**Figure 6:**
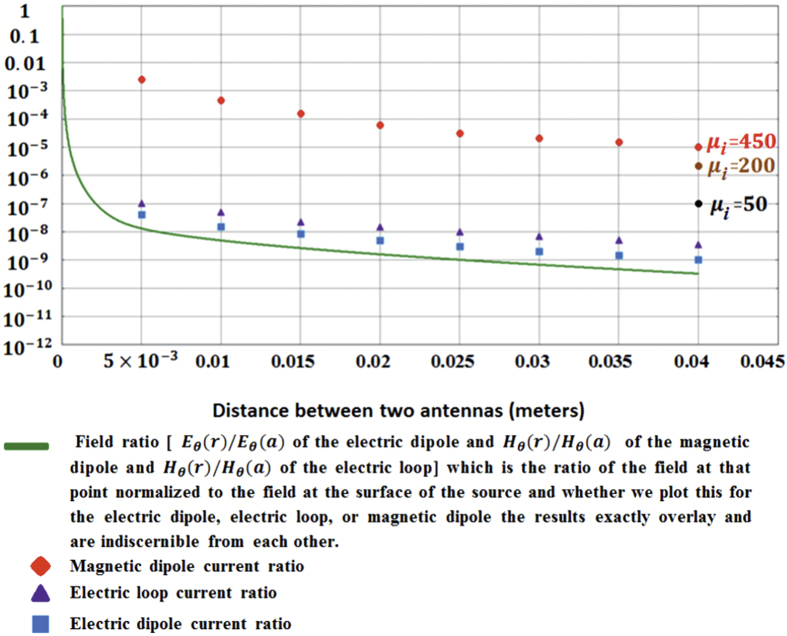
The fields of the electric dipole antenna, magnetic dipole antenna and the loop antenna normalized to the fields on their surface is plotted in the same figure as the induced current in each of these antennas normalized to the source current. The distance between the antennas has been change from 5 mm to 40 mm with 5 mm steps. The normalized fields of the three different antennas are the same but using a pair of magnetic dipole antennas results in a four order of magnitude improvement in the mutual coupling, eight order of magnitude improvement on power transfer, when compared to the conventional electric dipoles and loops.

Having defined all the relevant parameters we can calculate the current induced on a second antenna as a function of the current in the source antenna and the separation between them. For example, assuming there are two spheres (or loops) each of radius *a* = 10*μm*, separated from each other by a distance d, we let the distance d range from 5 mm to 4 cm in steps of 5 mms. The results, plotted as the ratio of the induced current to the source current, are shown as the symbols in Fig. 6. The advantage of the magnetic dipole antenna by about 4 orders of magnitude is startling. This corresponds to an 8 order of magnitude increase in power transmission, that is, +80 dB gain over the conventional alternatives.

To emphasize that the nature of the receiving antenna is as important as that of the transmitting antenna, we have also plotted in Fig. 6, as curves, the ratio of the principal field at the distance r = d to the maximum value of that field at the surface of the antenna r = *a*.
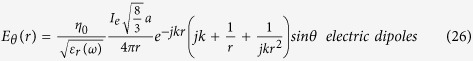

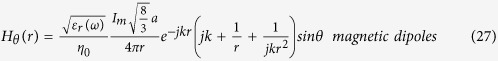

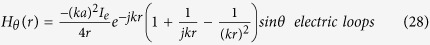


By examining this graph we again see the significant difference in the behavior of the mutually coupled magnetic dipoles. All the normalized fields lie on top of each other and 4 centimeters away the normalized field has dropped down by a factor of 3 × 10^−10^ compared to the field at the surface: −190 dB down. Electric dipole to electric dipole mutual coupling follows this same trend being slightly larger by a factor of 3, while the electric loop to electric loop coupling follows the same trend but stronger by about a factor of 10. The magnetic dipole coupling exceeds the field ratio by almost 5 orders of magnitude.

The efficiency of the magnetic dipole antennas are much higher than the other two alternatives (the electric dipole antenna and the electric loop antenna) because the magnetic material used for these antennas contributes to both the dipole moment and the input impedance. The material used for the magnetic dipole antenna calculations was assumed to have the permeability shown in Fig. 2. These magnetic materials already exist, and having a high *μ*_*initial*_ is an important factor which will result in having a strong magnetic conductivity. To illustrate the importance and role of the high permeability material, we have added two more data points at the distance of 4 cms showing that for *μ*_*initial*_ = 50 and *μ*_*initial*_ = 200 (with the same assumed resonant frequency) the advantage is not as significant as the *μ*_*initial*_ = 450.

An additional lesson from these calculations is that only looking at the rate of drop of a field component does not tell us the whole story because the field distribution in this space is dominated by the spherical spreading of the source field and the attenuation due to the medium whereas the way a receiving antenna can harvest energy out of this field is dominated by its self and mutual impedance properties.

## Discussion

In reference 1 the best antenna considered was the electric loop. The Fig. 6 plot of the induced current in the electric loop shows that the normalized induced current in the electric loop at 4 cm (far enough to get to the surface of the head from almost everywhere inside the brain) is stronger than the normalized induced current in the electric dipole and, at 3 × 10^−9^, it is about an order of magnitude stronger than the normalized field. But the current induced in the magnetic dipole is 2 × 10^−5^, almost five orders of magnitude larger than the field ratio. Why is this so?

The time domain response of an antenna to an ambient driving field depends on the quality or the “Q” of its resonance. Since the Q is inversely proportional to the damping, a very low Q antenna is strongly damped. This means that when driven by an ambient field such an antenna will respond, one to one, in direct proportion to the field strength it receives from the very beginning. However, a high Q antenna under the same ambient field will experience an ever increasing amplitude of oscillation until it reaches a steady state where the power dissipated matches the power input by the field.

The Electric dipoles at the considered size are strongly damped because of the near field direct loss in the body, and similarly, at this size, electric copper loops are also very much damped because of the required large current that dissipates a large amount of energy into their own conductivity. Therefore the response of electric dipoles and electric loops follow the field without any resonant amplification. But since true magnetic dipoles induce low body currents and require low metal currents to radiate they are much less damped and therefore develop a much higher current at resonance in response to the applied field.

Alternatively we can say that the excess loss of the metal antennas results in reduced receiving and transmitting cross sections when compared to the more efficient magnetic dipoles.

In summary, although the magnetic dipole antenna is fed by a loop, the effect of the permeable core is to change the character of the antenna from the short circuit of the electric loop, which has a high current, to an antenna that tends to an open circuit at resonance which as mentioned before is typical of dipoles, since dipoles are open circuits. Therefore its damping is dominated not by the copper loss but by the constitutive properties of its core. The ferromagnetic metals developed for the magnetic read-head industry on purpose combine high permeability with low damping and this is evidenced in the Lorentz line shape of their frequency dependent permeability; they are by design high Q materials.

As a “verification check” we performed an additional set of calculations in which we set the surrounding medium to free space. This change must remove the advantage of the magnetic dipoles. Indeed in that case we find that all of the antennas showed the exact same induced current.

Although the materials needed for the magnetic antennas already exist, the results obtained points out the importance of developing magnetic materials with high permeability and high resonant frequency. Given the small size of the antennas involved and the level of maturity of the magnetic read-head industry it can be anticipated that the development and production cost of these materials would not be an obstacle to their use. Magnetic read-head industry materials include multi-layers of “Permalloy” or other alloys with transition metals that have permeabilities in the hundreds and resonance frequencies as high as a few GHz. The magnetic properties of these materials can further be controlled by patterning their layers to control the formation of domains and alter the magnetic anisotropy^19^. Typical dimensions for these design features are in the 0.2 μm range^20^ fully compatible with an antenna structure in the assumed 10 μm size.

We have come to the conclusion that using coupled magnetic dipole antennas as microscopic links in an *in-vivo* telemetry system is a solution to the tissue damage problem caused by electric dipole antennas through SAR deposition and the electric loop antennas through heat conduction^21^,^22^,^23^. If we model the head as a sphere of approximately 5 cm radius, communication between a transmitting microscopic antenna anywhere inside the brain and an identical one used as a repeater node located just under the skull would derive the +80 dB in gain seen in Fig. 6 as compared to conventional antenna alternatives. Although full re-evaluation of the link budget for a telemetry system exploiting these antennas is yet to be performed we can state that microscopic neuron-by-neuron RF telemetry from within the brain is feasible to the extent that the antennas are no longer the bottleneck.

## Methods

Throughout this derivation we exploit the principle of duality inherent in Maxwell’s equations where to every conventional electric measurable (Voltage, Current) there is a dual magnetic measurable related through the transformations *E* *→* *H*, *H* *→* *−E*, *μ* ´ *ε*, Z_m_ = *Z*_*e*_/*η*^2^ The principal polarization fields radiated by electric and magnetic spherical dipoles of radius *a*, at a distance r, in terms of the current at the feed are respectively:
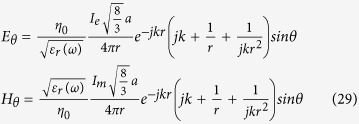


The quantity 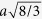 is known as the antenna height, *h*, for the spherical dipole that defines the voltage impressed across its feed by an incident electric field as *V*_*feed*_ = *E*_*inc*_*h* (and it is also the moment arm of its dipole moment.) For the electric dipole the current *I*_*e*_ is in Amps and is the conventional total current that would be crossing the equator of the sphere. For the magnetic dipole the current *I*_*m*_ is in Volts and is equal to the total polarization current crossing the equator of the sphere
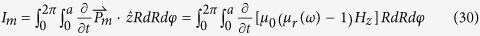


For simplicity we assume that the input impedance of the spherical antennas is closely modelled by the modal impedance of the first spherical wave mode (TM10 for the electric dipole and TE10 for the magnetic dipole.) For the small electric dipole this is given by equation 31.
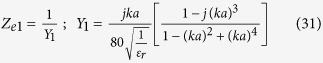


And for the magnetic dipole by its dual
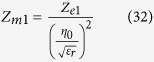


Then a self-consistent definition of the mutual Impedance is given by equation 33 where the numerator is the Reaction Integral between the two currents, that is, the inner product of the current in antenna 1 times the field antenna 2 produces on it. By the reciprocity of the Reaction Integral Z_12_ = Z_21_.
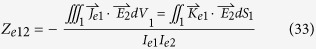


Where 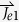 is the volumetric current and 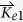 is the surface current and the numerator is showing both the volumetric current version and the surface current version of the reaction theorem.

Again, by duality:
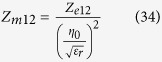


Assuming the spheres are small and far from each other the Reaction Integral simplifies by assuming a uniform field over the uniform current of the dipole and we get:
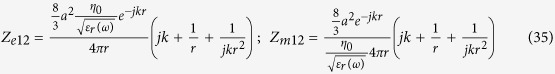


In practice, when the second sphere is farther than one diameter from the surface of the first sphere, the field is uniform enough that it can be extracted from the reaction integral as previously mentioned. Even though by definition the field wouldn’t be uniform closer than the mentioned distance, using this mutual impedance equation and coming exactly to the surface of the source antenna gives an answer that converges on the correct self-impedance. This convenient behavior is a result of the variational properties of the Reaction Integral in equation 33.

The mutual impedance of the electric loop has been obtained using two different methods. The first method is finding the mutual inductance and the second method which also serves as a verification check is calculating the reaction integral (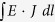) by replacing the loop with a curvilinear square of equal area to make the calculation of the integral very easy.

The mutual inductance found from the first method and the mutual impedance are as follows, where ‘r’ is the distance between the antennas. (As alluded to above, the mutual impdance tends to the self impedance at r = *a*.)
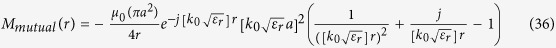

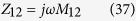

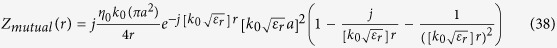


For the second method we will perform the reaction integral of *E*_*φ*_ times the circulating current in the loop and that would give us the mutual impedance. A good approximation is to replace the the loop with a curvilinear square of equal area. Now the reaction integral is trivial.
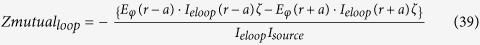


After simplifying we will have:
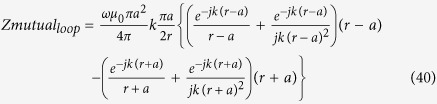


And the final result is as follows:
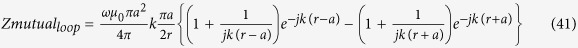


The results obtained from the two different methods are very close.

### Finite difference time domain (FDTD) simulation approach

The results shown in Fig. 6, show a dramatic advantage in the case of true magnetic antennas. Even though the closed-form expressions for analyzing this problem have been derived following classic approaches in antenna theory, the results are almost “too good to be true.” Therefore it is appropriate to spot check the result by an alternate method. The alternative to the analytic formulation is a numerical simulation approach. Such an approach is not without its own difficulties.

In many ways the problem addressed here resembles the problem of the scattering of light from plasmon resonant sub-wavelength particles. In that case, for particles of the order of 10 nm in diameter the scatterer is of the order of two hundredths of a wavelength. In our case an antenna 20 microns across at 2 GHz in the body dielectric is less than one thousandth of a wavelength. To faithfully model such small particles and correctly simulate the resonant behavior with frequency domain Volume Integral Equation methods or Finite Element methods requires an extremely fine mesh and the inversion of a matrix that at resonance has a nearly zero determinant. Special methods for dealing with this sub-wavelength problem have been devised, but are not usually implemented in the commercially available software.

Because all we want to do is spot check the result, a reliable brute force approach is to solve the problem using the Finite Difference Time Domain (FDTD) method. By recognizing that the advantage of the magnetic antenna occurs for every separation distance considered, we can choose a case where two antennas are relatively close and thus examine the case of strong near field coupling. A best case for the electric dipole antenna is chosen where the average body dielectric is more heavily biased towards fat, thus assuming a dielectric constant near 2 GHz of 56 and an equivalent conductivity of 1.07 S/m. The antennas are 10 *μ*m in radius, 56 *μ*m center to center apart. The closed form expressions for this case yield the induced magnetic current on the second particle to be of the order of 0.67 (−3.47 dB) that of the first whereas for electric dipoles it is 0.06 (−24 dB) of the first.

**Figure 7:**
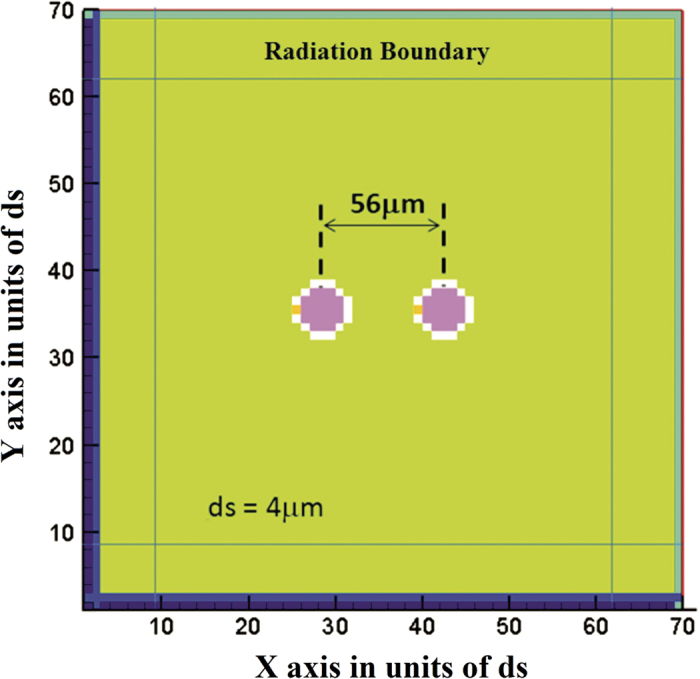
Top view of the FDTD domain used to analyze two coupled micro-antennas

We choose an FDTD domain discretized uniformly with dx = dy = dz = ds = 4 *μ*m, and dimension 70 ds by 70 ds by 70 ds. This domain is small enough to run 2.5 million time steps in 2 hours on a 16 core CPU. Such a small domain (*λ*/100 on the side) could not be used to calculate far field radiation from these antennas. However since it is 14 antenna diameters across it is large enough to accommodate the quasi-static near field that dominates the behavior of these antennas. Even at this small size, the discretization is coarse as illustrated in Fig. 7 where the two antennas are shown as seen from above.

A closed flux path formulation has been employed to maximize symmetry; as in the PEC blocks mentioned in the work done by Z. Zhang and R. E. Diaz^24^. Because of the coarse discretization, even though the antennas are spheres 2.5 ds in radius their actual physical size may appear to be larger by one discretization cell (24 microns rather than 20 Microns). Similarly, it is evident that since the conducting bands have been made thick for the same reason, the actual equivalent separation between the antennas for near field coupling may be one cell shorter, or 52 microns instead of 56 microns. Such deviations of up to 20% in linear dimension are considered to be slight for the purpose of this computation because the difference in coupling between these antennas according to the closed form model is of the order of 20 dB, that is, a factor of ten in induced current. Since we have mentioned that the actual physical sizes may appear to vary by one discretization cell we have found the closed form expressions for different sizes and distances and the results are very close ranging from −3.75 dB to −3.29 dB for the magnetic dipoles and −23.1 dB to −24.6 dB for the electric dipoles, showing that the deviations in size are in fact too small to make a significant difference in the coupling.

Since the principal loss mechanism for the case of the magnetic antennas is the permeable material, the spherical core is assumed to have a relative permeability of 300-j300. That would be the equivalent of using a laminate of 0.75*μ*m CZN ferromagnetic alloy layers alternating with 0.5*μ*m layers of insulator (SiO_2_). In the case of the electric antennas the principal loss mechanism is the current induced in the body medium; thus the core for that case was assumed to be a perfect electric conductor. The core is shown as the pink region in the center of each sphere.

The antennas are made to resonate by wrapping them around the equator with a perfectly conducting “belt” (electrically conducting for the magnetic dipole, magnetically conducting for the electric) that has a gap of one discretization cell on one side of the antenna (the white region surrounding the core). The gap (orange squares in the figure) is filled with a low loss material of high permittivity (magnetic antenna) or permeability (electric antenna) such as to induce resonance close to 2 GHz. Although this construction is less realistic than that assumed in the paper it ensures that the comparison between electric antennas and magnetic antennas is as fair as possible by minimizing their differences.

**Figure 8:**
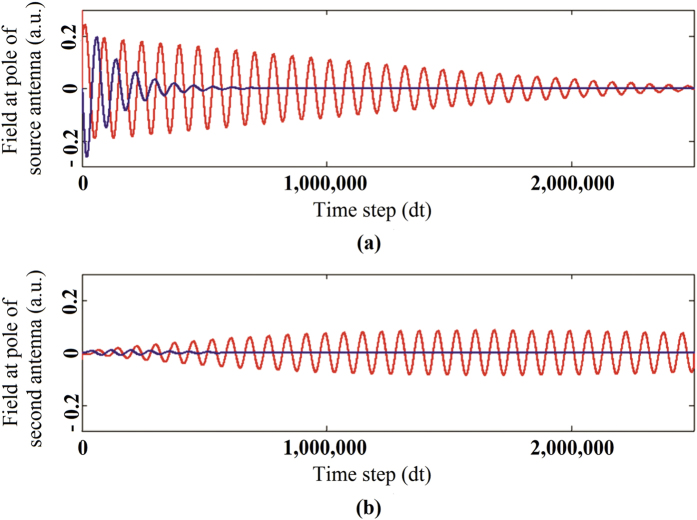
Time domain history of the interaction between the antennas when the first one is excited by an impulse. 8(**a**) current in the source antennas where blue shows the electric dipole and red shows the magnetic dipole. 8(**b**) current in the second antennas where blue shows the electric dipole and red shows the magnetic dipole.

The antenna on the left is excited by driving a time domain field impulse from one end of the gap in the belt to the other. In the case of the magnetic antenna this is an electric field impulse. In the case of the electric antenna this is a magnetic field impulse. Using a magnetic field impulse on a perfectly magnetically conducting belt as the source for the electric antenna also eliminates any question about whether or not the electric antenna source at its feed could be short circuited by the body dielectric. Figure 8 shows the results of the time domain simulation.

The field produced just above the pole of the antennas is measured as a function of time. Because of the continuity of the total Maxwell current density this field value is a direct measure of the current “flowing” through the antenna. The upper figure shows the current in the source antenna while the lower figure shows the current in the second antenna. The difference between a magnetic antenna and an electric antenna in the body medium is evident. The source antenna current dies off exponentially for the electric antenna (blue in the figures) whereas for the magnetic antenna (red in the figures) the drop at the source is a combination of a weak exponential attenuation and attenuation due to the transfer of energy to the second antenna.

**Figure 9:**
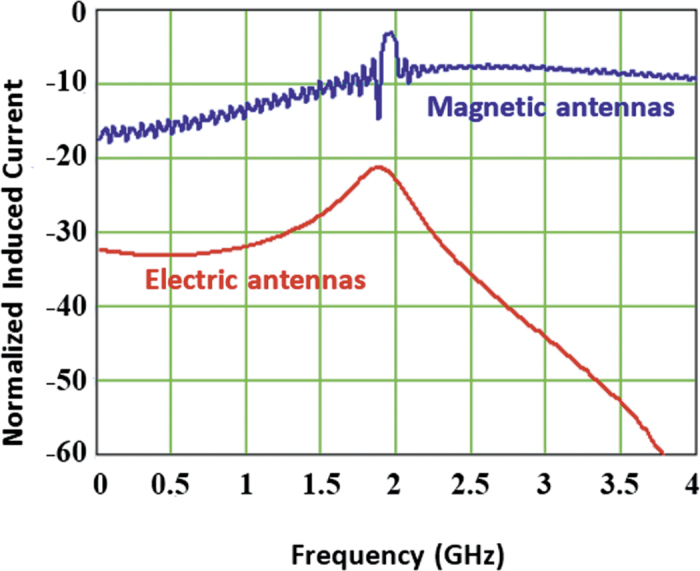
Frequency domain induced current on the second antenna

That this is what is happening, is evident from the second antenna current where we see that for the magnetic antenna the oscillation is still going strong after 2.5 million time steps (only 17 ns of real time has been simulated). Taking the Fourier Transform of these currents and expressing the current in the second antenna relative to the first we obtain the result shown in Fig. 9. The current induced on the magnetic antenna is −3 dB down from the source whereas the current induced on the electric antenna is −21 dB down which shows results similar to those obtained from the closed form calculations.

## Additional Information

**How to cite this article**: Yousefi, T. and Diaz, R. E. Pushing the limits of radiofrequency (RF) neuronal telemetry. *Sci. Rep.* **5**, 10588; doi: 10.1038/srep10588 (2015).

## Figures and Tables

**Table 1:** 

**Antenna**	**Electric Loop antenna**	**magnetic dipole**
Radiation Resistance (electric or magnetic)	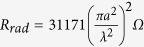	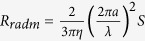
Material Resistance (electric or magnetic)	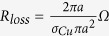	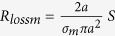
Efficiency	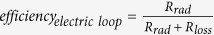	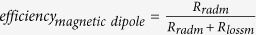
Input Impedance at resonance (Ω)